# Review: Vaspin (SERPINA12) Expression and Function in Endocrine Cells

**DOI:** 10.3390/cells10071710

**Published:** 2021-07-06

**Authors:** Patrycja Kurowska, Ewa Mlyczyńska, Monika Dawid, Małgorzata Jurek, Dominika Klimczyk, Joelle Dupont, Agnieszka Rak

**Affiliations:** 1 Laboratory of Physiology and Toxicology of Reproduction, Institute of Zoology and Biomedical Research, Jagiellonian University in Krakow, 31-387 Krakow, Poland; patrycja.kurowska@doctoral.uj.edu.pl (P.K.); ewa.mlyczynska@doctoral.uj.edu.pl (E.M.); monika.dawid@student.uj.edu.pl (M.D.); malgorzata99.jurek@student.uj.edu.pl (M.J.); d.klimczyk@student.uj.edu.pl (D.K.); 2 Unité Physiologie de la Reproduction et des Comportements, UMR85, INRAE, 37380 Nouzilly, France; joelle.dupont@inrae.fr

**Keywords:** vaspin, endocrine cells, hypothalamus, pituitary, adipose tissue, pancreas, thyroid, ovary, placenta, testis

## Abstract

Proper functioning of the body depends on hormonal homeostasis. White adipose tissue is now known as an endocrine organ due to the secretion of multiple molecules called adipokines. These proteins exert direct effects on whole body functions, including lipid metabolism, angiogenesis, inflammation, and reproduction, whereas changes in their level are linked with pathological events, such as infertility, diabetes, and increased food intake. Vaspin-visceral adipose tissue-derived serine protease inhibitor, or SERPINA12 according to serpin nomenclature, is an adipokine discovered in 2005 that is connected to the development of insulin resistance, obesity, and inflammation. A significantly higher amount of vaspin was observed in obese patients. The objective of this review was to summarize the latest findings about vaspin expression and action in endocrine tissues, such as the hypothalamus, pituitary gland, adipose tissue, thyroid, ovary, placenta, and testis, as well as discuss the link between vaspin and pathologies connected with hormonal imbalance.

## 1. Introduction

Communication between tissues and the coordination of body function is regulated by both the endocrine and nervous systems [1]. Endocrine glands secrete hormones, chemical signals that are distributed throughout the body by blood [2]. By affecting every tissue, the importance of hormones for the homeostasis of the human body is underscored. The hypothalamus and pituitary are central endocrine glands, and multiple organs are peripheral endocrine glands, including the pancreas, thyroid, adrenal, parathyroid, gonads, placenta, thymus, and pineal glands [3]. Briefly, the hypothalamus regulates the anterior pituitary, water balance, food intake, reproductive system, and generation and regulation of circadian rhythms [4]. Pituitary hormones influence growth, reproduction, metabolic homeostasis, and blood volume regulation [5]. The thyroid gland produces thyroxine (T4) and triiodothyronine (T3), which impact gluconeogenesis and lipogenesis and participate in thermoregulation [6]. The pancreas secretes insulin and glucagon, which play a major role in carbohydrate and lipid metabolism and energy storage [7]. The ovaries, testes, and placenta synthesize steroid hormones, such as oestradiol (E2) and progesterone (P4), which are essential to ovulation, sperm production, and pregnancy maintenance [8]. Interestingly, white adipose tissue (WAT) may be the largest endocrine tissue in the human body, secreting more than 1000 potential cell-signaling mediators [9], called adipokines, including cytokines, growth factors, hormones, chemokines, and proteins [10]. Adipokines are involved in multiple functions in the organism, including glucose homeostasis, lipid metabolism, angiogenesis, inflammation, and blood pressure regulation, as well as reproduction [10]. Vaspin is a newly described adipokine with an insulin sensitizing effect [11] and inhibitory impact on food intake [12]. Moreover, the imbalance of hormonal homeostasis leads to many disorders, including acromegaly, Addison’s disease, diabetes, diabetes mellitus, Hashimoto’s disease, and infertility. For example, higher plasma vaspin levels were described in the most common ovarian pathology, polycystic ovarian syndrome (PCOS) [13].

Therefore, in this review, we collected data about vaspin, focusing on its expression and function in endocrine cells, including the hypothalamus, pancreas, thyroid gland, adipose tissue, ovaries, placenta, and testes, as well as the link between vaspin and different disorders connected to endocrine imbalance.

## 2. Vaspin Structure and Expression

Vaspin is an adipokine belonging to the serpin family, thus also named SERPINA12 [11], with targets in kallikrein 7 and 14 that participate in skin desquamation [14,15]. Interestingly, vaspin–heparin interactions accelerate the inhibition of kallikrein 7 [16]. This adipokine features all typical structural domains of native serpins with three β-sheets, nine α-helices, and a flexible reactive center loop (RCL), containing a protease recognition sequence on the top [17]. In its amino acid sequence, vaspin exhibits 40.5% homology with α1-antitrypsin [11]. Rat, mouse, and human vaspins are composed of 392, 394, and 395 amino acids, respectively. Furthermore, human and rat vaspin amino acids exhibit 61.5% identity, whereas human and mouse share 62.6% [14]. Rats, mice, and human vaspin presents a signal peptide on the N-terminal domain; additionally, in mice N-terminal Edman sequencing revealed that the vaspin protein begins at the Leu-21 residue, and the vaspin RCL comprises residues G364–P381 [11]. The side chain with a non-charged amino acid at position P14 within the hinge region of the RCL indicate that vaspin belongs to the serpins. A cleavage site between methionine 378 (P1) and glutamate 379 (P1′) in the RCL sequence was experimentally verified, which likely determines protease specificity [14]. This protease specificity is also regulated by arginine 302, which enables vaspin recognition by kallikrein 7, whereas P1 glutamate 379 is a major limiting factor for vaspin inhibitory activity [18]. Interestingly, in the native structure, residues glutamate 379 (P1′) and threonine 380 (P2′) of the RCL interact with arginine 302 of β-sheet C via a salt bridge and a water-mediated hydrogen bond, respectively. In a vaspin structure, by manipulation of the RCL sequence by introducing a serine at P1′ (glutamate 379S) and an artificial disulfide bridge in proximity to the scissile bond (aspartate 305C/ valine 383C), the flexibility of the C-terminal part of the RCL was enhanced. As described previously, human vaspin is a glycoprotein with three predicted glycosylation sites in the N-terminus at asparagine residues. Two of them are located in close proximity to the RCL and near domains connected to conformational changes regulating serpin inhibition mechanisms. Interestingly, glycosylation of vaspin does not hinder inhibition of the target protease kallikrein 7 [19]. In rat and mice vaspins there is a lack of the two glycosylation sites near the RCL, but an additional predicted site near the bottom of helix C on the back side of the serpin molecule was observed [20]. A 45 kDa vaspin protein, observed in mouse and human adipose tissue after Western blot protein analysis, revealed the presence of two bands at 45 and 50 kDa in the serum, indicating the processing or cleavage of vaspin by unknown serine proteases [11]. Moreover, as was described, the vaspin-kallikrein 7 complex has an apparent molecular weight of 70 kDa [14]. Interestingly, glycosylation has a significant effect on vaspin molecular weight, e.g., human unglycosylated vaspin migrates at 45 kDa, whereas glycosylation increases the molecular weight by around 7.5 kDa [19]. In addition, lower migrating vaspin bands may be connected to vaspin-derived peptides from the N terminal, which are released as a reason for protease cleavage [21]. These cell-penetrating peptides have biological effects connected to preadipocyte proliferation, and also present an additional aspect of vaspin action [21].

Vaspin is encoded by the *SERPINA12* gene that is present on the long arm of chromosome 14 (14q32.1) in humans and consists of 1236, 1242, and 1245 nucleotides in rats, mice, and humans, respectively [11]. A growing amount of evidence indicates that single nucleotide polymorphisms in the *vaspin* gene influence its serum concentration. A decrease in the vaspin circulating level was observed in the rs61757459 polymorphism due to an early stop codon, resulting in a truncated protein that is lysosomally degraded because of misfolding [22]. Conversely, the rs77060950 polymorphism, present in a Japanese population, exhibited significantly higher serum levels by regulating *vaspin* transcriptional activity [23].

Its expression was found in visceral (VAT) [11] and subcutaneous adipose tissue (SAT) [24], the skin [25], liver, pancreas [26], placenta [27], stomach, cerebrospinal fluid, hypothalamus [12], and ovaries [28]. The expression level of this adipokine is dependent on the tissue. In mice, was expressed in the brain, skin, and liver; moderate in adipose tissue; and had no expression in kidneys or muscles [29]. Vaspin content was also observed in serum, with human vaspin levels ranging from 0.18 to 1.55 ng/mL [30]. Similarly, in pigs, vaspin was around 1 ng/mL [28]. Significantly lower vaspin levels were observed in the serum of underweight children compared to the control [31], whereas gene expression of *vaspin* is upregulated when body mass increases [24]. The literature described several vaspin regulators. In rats and humans, VAT expression and serum concentration increased with insulin resistance, obesity, and leptin elevation and decreased with weight loss [11,23,24,32,33]. *Vaspin* was upregulated in liver and brown adipose tissue (BAT) after exposure to a high-fat diet [29]. Collectively, these findings suggest that vaspin is a compensatory molecule in obesity and insulin resistance [34]. Interestingly, interactions between vaspin and different hormones were observed. Insulin increased adipose tissue vaspin levels in Otsuka Long-Evans Tokushima Fatty (OLETF) rats [11], and insulin-sensitizer metformin increased *vaspin* mRNA in the gonadal adipose tissue of rats [35]. Conversely, metformin decreased serum vaspin levels in patients with PCOS [13]. Furthermore, sexual dimorphism in vaspin levels was observed, in which there was a significantly higher vaspin content in women than men [35], indicating that steroid hormones impact vaspin expression. Another confirmation of these findings is that vaspin serum levels were elevated in women using oral contraceptives [36]. Furthermore, in children, elevated circulating vaspin concentrations were observed in girls and were stimulated with the puberty stage [26]. Moreover, vaspin levels changed during the day, with higher circulating vaspin before a meal than after a meal; the highest amount was observed at night, which is reciprocal to insulin levels [37].

## 3. Vaspin Receptor GRP78 and the Mechanism of Vaspin Action 

Vaspin binds to the cell surface to a 78 kDa glucose-regulated protein (GRP78), also known as the heat shock protein family A member 5 (HSPA5) or binding immunoglobulin protein, which is recruited from the endoplasmic reticulum (ER) to the plasma membrane under ER stress [38]. GRP78 is a 78 kDa protein and its structure is divided into three domains: a 44 kDa ATP binding domain at the amino (N) terminal (ABD), a 20 kDa substrate (polypeptide) binding domain (SBD) at the carboxyl (C)-terminal, and a 10 kDa domain in the C-terminal tail with unknown function [39]. GRP78 shares 60% homology with the HSP70 family, with the conservation of ABD and SBD domains [40], and its protein consists of 654 amino acids in humans and 655 in mice [41]. 

In humans, the gene responsible for encoding GRP78, *HSPA5*, was found on chromosome 9 and consisted of 4532 nucleotides grouped in eight exons [42]. Human *GRP78* mRNA is expressed in the brain, endometrium, placenta, ovaries, spleen, testes, and thyroid. The *Grp78* gene in mice was localized on chromosome 2, and expression was noted in the placenta, ovaries, spleen, testes, thymus, and brain. Rat *Grp78* is found on chromosome 3 with expression in the brain, spleen, testes, and thymus, whereas in pigs, it is on chromosome 1, and its expression was detected in the ovaries, spleen, and adipose tissue [41]. In the functional *GRP78* gene, short domains in the hydrophobic region are highly conserved with HSP70 from humans, *Drosophila*, *Xenopus*, yeast, and *Escherichia coli* [42].

Intracellular expression of the GRP78 protein was found in the ER lumen, but the protein was also present on the cell surface; this localization gives it the possibility to act as a receptor [43]. Some factors, including hormones, may regulates *Grp78* expression; in rat ovaries, it is upregulated by prostaglandin (PG) PGF2α [44] and in cows by follicle-stimulating hormone (FSH) [45]. Additionally, leptin induced GRP78 expression in neuronal cells [46]. The main function of GRP78 is the translocation of polypeptides throughout the ER membrane, maintenance of intracellular Ca^2+^ homeostasis, and regulation of the process of Ca^2+^ efflux from the ER to mitochondria; however, on the cell surface, its purpose is cell proliferation and survival [47]. It also functions in the physiology and pathology of the female reproductive tract, including follicular, corpus luteum (CL), oviduct, uterus, embryo, and preimplantation development [48]. Cell-surface GRP78 is also a major autoantigen in ovarian cancer [49] and inhibits angiogenesis of endothelial cells [50]. Interestingly, in eIF2α-mutant mice that developed severe obesity associated with a reduced metabolic rate, GRP78 effectively reduced insulin levels and participated in adipogenesis and glucose homeostasis in adipose tissues when fed a high-fat diet [51]. GRP78 is a master regulator in the ER and changes in its expression or activation are associated with cancer, and cardiovascular and neurodegenerative disease [41]. Overexpression of GRP78 increases the proliferation, migration, and invasion, as well as the percentage of cells in the S phase of the cell cycle, of pancreas cancer cells [52]. 

**Figure 1:**
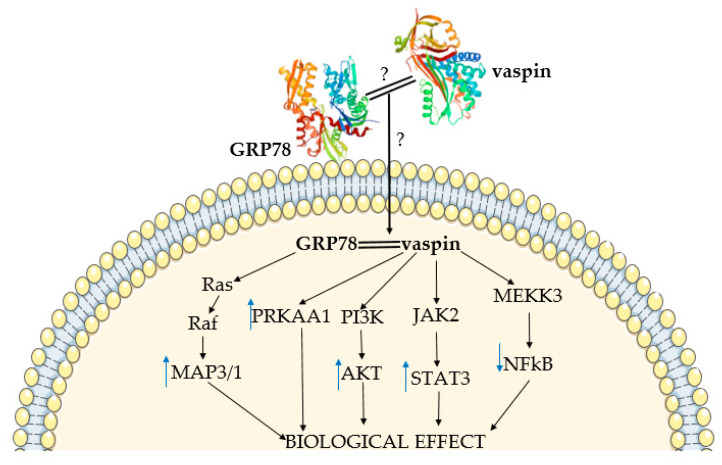
Summary of mechanism of vaspin action. Vaspin (10.2210/pdb4IF8/pdb) and GRP78 structures (10.2210/pdb3LDL/pdb) based on the Protein Data Bank. PRKAA1-AMP-activated kinase; MAP3/1-mitogen-activated kinase; AKT-protein kinase B; STAT3-Janus kinase; NFkB- nuclear factor kappa B; ↑—increase; ↓—decrease; ?—data limitation.

Furthermore, previously published data showed that vaspin binds GRP78, as well as transduces intracellular signaling via multiple kinase pathways. Interestingly, Nakatsuka et al. showed that vaspin binds GRP78 via the helical domains in the N-terminus and not by the RCL region [38]; the binding site in GRP78 remains unknown. Nevertheless, the mechanism of signal transduction into the cell is also unknown and probably depends on high GRP78 affinity for negatively charged cell membrane phospholipids [53]. Vaspin also binds to phospholipids, which play a major role in membrane trafficking [54]. Additionally, a previous study described positive vaspin interaction with protein kinase B (AKT) and AMP activated kinase (PRKAA1) phosphorylation [38]. Moreover, vaspin increased the AKT phosphorylation protein level in pancreatic islets, with a negative effect on nuclear factor kappa B (NFκB) [55]. Vaspin might attenuate the cytokine-induced expression of adhesion molecule genes by inhibiting NFκB, following AMPK activation [56]. Additionally, vaspin increases endothelial nitric oxide synthase activity through Janus kinase (STAT3) activation in vascular endothelial cells [57]. By activating the GRP78 and mitogen activated kinase (MAP3/1) pathway, vaspin regulates porcine ovary function [58,59]. Using the MAPK/p38 pathway, vaspin protects human osteoblasts from apoptosis [60]. Interestingly, together with GRP78, vaspin was co-localized with MTJ1 protein in liver, and with a voltage-dependent anion channel (VDAC) in human aortic endothelial cells. Vaspin and GRP78/MTJ1 complex interactions lead to intracellular signaling cascades in hepatocytes, including activation of AKT and AMPK. On the other hand, in endothelial cells, vaspin exerts anti-apoptotic effects by preventing Kringel5 from binding to the GRP78/VDAC complex, as well as inhibiting the increase of intracellular Ca^2+^ levels [20]. Interactions between vaspin and GRP78, as well as with different kinase pathways, indicates its important role in regulating cell function (Figure 1).

## 4. Vaspin Expression and Action in the Hypothalamus and Pituitary

**Figure 2:**
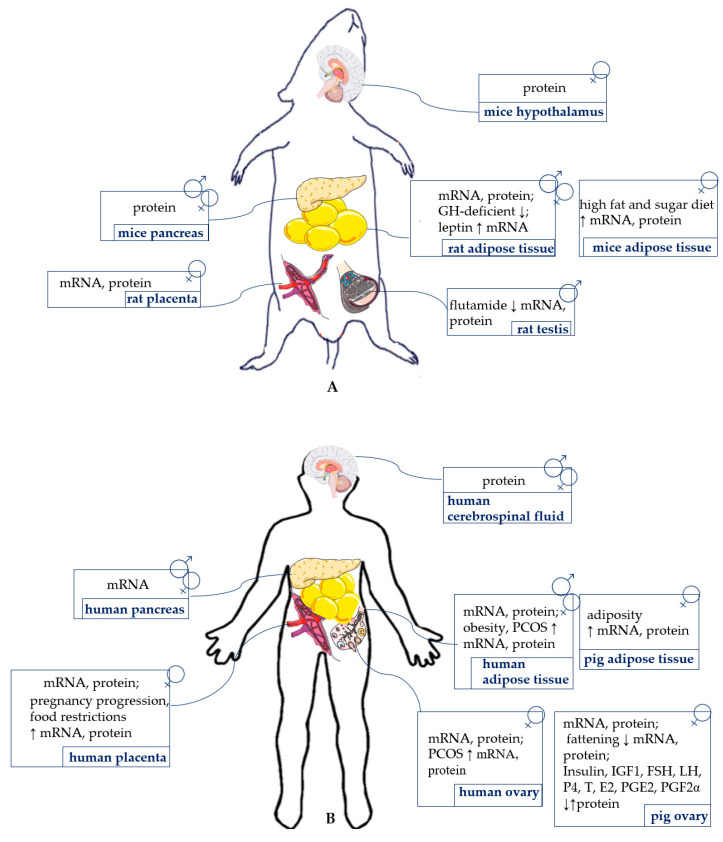
Vaspin endocrine cell expression and its regulation in rodents (**A**), and human and domestic animals (**B**). PCOS—polycystic ovarian syndrome; GH—growth hormone; IGF1—insulin-like growth factor type 1; FSH—follicle-stimulating hormone; LH—luteinizing hormone; P4—progesterone; E2—oestradiol; T—testosterone; PG—prostaglandin; ↑—increase; ↓—decrease.

**Figure 3:**
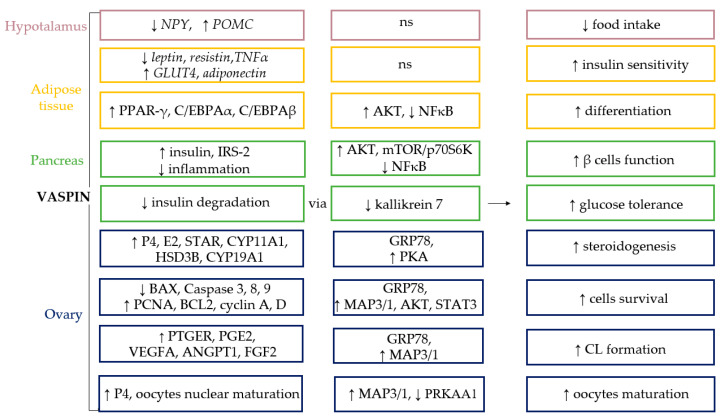
Vaspin function in endocrine cells. NPY—neuropeptide Y; POMC—proopiomelanocortin; TNFα—tumor necrosis factor; GLUT4—glucose transporter-4; PPAR-γ—peroxisome proliferator-activated receptor γ; IRS-2—insulin receptor substrate 2; C/EBPA—CCAAT/enhancer-binding protein α (C/EBPα) and β (C/EBPβ); P4—progesterone; E2—oestradiol; STAR—steroidogenic acute regulatory protein; CYP11A1—cytochrome P450 family 11 subfamily A member 1; HSD3B—hydroxy-delta-5-steroid dehydrogenase; CYP19A1—cytochrome P450 family 19 subfamily A member 1; BAX—BCL2-associated X; PCNA—proliferating cell nuclear antigen; BCL2—B-cell lymphoma 2; PTGER—prostaglandin E receptor; PG—prostaglandin; VEGFA—vascular endothelial growth factor; ANGPT1—angiopoietin 1; FGF2—fibroblast growth factor 2; AKT—protein kinase B; NFkB—nuclear factor kappa B; GRP78—78 kDa glucose-regulated protein; PKA—protein kinase A; MAP3/1—mitogen activated kinase; STAT3—Janus kinase; PRKAA1—AMP-activated kinase; CL—corpus luteum; ↑—increase; ↓—decrease; ns—no study.

The protein expression of vaspin was detected in the whole hypothalamus of the diabetic (db/db) and C57BL/6 mice models by Western blot, as well as in cerebrospinal fluid of humans [12] (Figure 2). This adipokine reduces food intake and lowers blood glucose levels in mice, but this effect was reversible after one day without vaspin injection [12]. Additionally, vaspin decreased temporary food intake, as well as led to a higher metabolic rate in rats [61]. Both results indicate a quick, ad hoc regulation of food intake by vaspin. Moreover, as shown in rats, central administration of vaspin triggers anorectic pathways, decreasing mRNA expression of neuropeptide Y (*NPY*) and increasing proopiomelanocortin (*POMC*), which mediates nutritional suppression [62]. This mechanism clearly indicates that vaspin, similar to well-described leptin, may reduce food intake in mammals (Figure 3). Moreover, other studies indicated higher vaspin expression in the hypothalamus of rats with a high-fat diet and suggested that the administration of vaspin to the third ventricle of the brain inhibits hepatic glucose production and increases insulin sensitivity in rats with insulin resistance via the dorsal vagal complex to the hepatic branch of the vagus nerve. This effect is linked to downregulated mRNA and protein hepatic G6Pase (glucose 6-phosphatase), and phosphoenolpyruate carboxykinase expression levels and elevated hepatic insulin receptor, insulin receptor substrate-1, and AKT kinase [62]. Moreover, other data suggest that the concentration of vaspin in serum may also modulate eating behavior in humans, but single nucleotide polymorphisms associated with vaspin concentrations show no connection with eating behavior [63]. Vaspin may decrease food intake by increasing insulin levels, which has an anorectic effect in the brain by inhibiting its degradation via kallikrein 7 [14]. Vaspin action may also depend on its interactions with GRP78. Briefly, high-fat-diet-fed rats were characterized by hypothalamic ER stress. GRP78 overexpression specifically in the ventromedial nucleus of the hypothalamus reverted this effect and obese phenotype [64]. Additionally, stereotaxic treatment with adenoviruses harboring GRP78 reduced hypothalamic ER stress and reversed high-fat-diet-induced obesity in rats, which is probably associated with an increased BAT thermogenesis [65]. The obtained data indicate that an increase in obesity vaspin levels may play a compensatory role by decreasing food intake. This adipokine could possibly be used in the future as a slimming drug.

Research on vaspin expression in the pituitary gland is clearly limited. Changes in pituitary function have been shown to modify the levels of vaspin in the body. It was found, inter alia, that rats with pituitary dissection displayed lower levels of *vaspin* mRNA compared to control rats in WAT [35]. Furthermore, the expression of *vaspin* mRNA was decreased in growth hormone (GH)-deficient rats compared to control rats, indicating that GH deficiency reduced vaspin levels [35]. Research indicates that pituitary dysfunction modifies vaspin levels, e.g., the level of FSH produced by the pituitary gland negatively correlates with serum vaspin concentrations [66]. The pituitary gland is the overriding endocrine organ. The aforementioned studies clearly show that the hormones produced by the pituitary gland regulate the level of vaspin, indicating that this hormone may be necessary for organ homeostasis. Future research is necessary to understand the influence of vaspin on the secretory function of pituitary cells and to study its expression in this structure.

## 5. Vaspin Expression and Action in Adipose Tissue

For the first time, vaspin mRNA and protein expression was detected in VAT in OLETF rats [11]. Its expression was also shown in the SAT of rats and in human adipocytes [24], as well as in BAT in mice [29]. Interestingly, *vaspin* expression was detected only in some patients, but was more often noted in obese patients [24]. Using an immunohistochemical technique, vaspin expression was demonstrated in the WAT of pigs [28]. *Vaspin* levels in BAT were higher in mice exposed to high-fat and -sugar diets and in WAT in mice exposed to low temperatures [29], indicating the role of vaspin in thermogenesis. Interestingly, *vaspin* and *GRP78* were more greatly expressed in the SAT of obese patients, and its secretion was elevated in in vitro cultures of adipocytes from obese people [67]. Moreover, in obese Korean women who had abdominal gynecological surgery, a decrease in *vaspin* expression was observed in the SAT, accompanying an increase in the VAT [68]. Additionally, vaspin and GRP78 levels depend on fattening in pigs, with significantly higher expression of both protein and mRNA levels detected in the adipose tissue of fat Meishan pigs compared to lean Large White pigs [28], which was confirmed by analogical changes in vaspin serum levels [69]. Furthermore, in rats on a fasting diet, leptin increased *vaspin* levels in WAT [35]. Furthermore, *vaspin* mRNA expression in WAT was significantly downregulated in GH-deficient rats, suggesting a positive correlation between vaspin and GH levels [35] (Figure 2) and indicating that vaspin is positively connected with organism growth. Research also showed that *vaspin* mRNA levels in WAT were significantly downregulated in rats with hyperthyroidism and significantly increased in rats with hypothyroidism when compared to the control. Thyroid dysfunction is frequently associated with metabolic changes and affected body mass, indicating that hypothyroidism is connected with weight loss [35].

Vaspin at 1 mg/kg weight has been shown to have insulin-sensitizing effects in the WAT cells of mice with a negative effect on glucose concentrations [11]. Furthermore, in visceral mesenteric and subdermal adipose tissue, vaspin decreased the expression of *leptin*, *resistin*, and tumor necrosis factor (*TNFα*) but increased the expression of glucose transporter-4 (*GLUT4*) and *adiponectin*. Moreover, after the administration of vaspin, mRNA expression levels of genes associated with metabolic syndrome (MetS) (*GLUT4, resistin, adiponectin* and *leptin*) returned to normal levels in obese rats’ WAT [11]. The inhibitory effect of vaspin on the expression and secretion of pro-inflammatory cytokines interleukin 6 or TNFα has been demonstrated in the 3T3L-1 cell line [70]. Moreover, vaspin in high doses (100 and 200 ng/mL) increased the mRNA and protein expression of transcription factors, such as peroxisome proliferator-activated receptor γ (PPAR-γ), and CCAAT/enhancer-binding protein α (C/EBPα) and β (C/EBPβ), leading to the differentiation of 3T3-L1 preadipocytes and lipid accumulation [71]. Conversely, no effect of vaspin was observed by Zieger et al. [70]. Additionally, vaspin decreased hypertrophy of adipocytes under a high-fat diet [38]. As a mechanism of vaspin action in the 3T3-L1 cell line, the authors described the phosphorylation of AKT kinase, as well as decreasing NFkB levels [70] (Figure 3). The presented data collectively indicate that vaspin plays a protective mechanism in obesity, characterized as a chronic inflammation connected to insulin resistance by its insulin-sensitizing and anti-inflammatory effects.

## 6. Vaspin Expression and Action in the Pancreas

Vaspin, like other hormones from the adipokine group, is involved in the regulation of glucose metabolism. Thus far, its expression and action have been demonstrated in human and rodent pancreatic islets [14,26,55] (Figure 2). Studies on the rat insulinoma cell line (INS-1) have shown that upon treatment with a dose of vaspin (80–320 ng/mL), the amount of mRNA for the insulin receptor substrate 2 (*IRS-2*) and the expression of IRS-2 total protein were higher compared to the group treated with palmitic acid, whereas its phosphorylated forms decreased. Additionally, vaspin stimulated the secretion of insulin by INS-1 cells, and this effect was mediated through activation of kinase AKT and the mTOR/p70S6K-signaling pathway [55]. Hence, vaspin action is complex, not only improving the insulin resistance of islet β cells, but also improving the insulin secretion from INS-1 cells. The inhibition of inflammation in pancreatic cells by vaspin was achieved through the downregulation of NFκB. It is a well-known fact that type 2 diabetes (T2DM) is linked with chronic inflammatory response mediated by inflammatory factors. Thus, high cytokine levels were noted in pancreas islets of T2DM patients, leading to damage in pancreatic β cell activity and β cell failure. Therefore, vaspin may improve the functions of β cells [55]. Moreover, vaspin reversed the inhibitory effect of palmitic acid on the proliferation of INS-1 cells [55], which may also improve its secretory activity. Next, vaspin may also influence the stability and half-life of insulin in circulation. In db/db mice, as in the control group, an increase in blood insulin levels was achieved after treatment with vaspin. Vaspin, due to its serpin activity, can inhibit the degradation of insulin by kallikrein 7, which cuts insulin within the A and B chain. Vaspin was co-localized with kallikrein 7 in murine pancreatic islets, as the vaspin-kallikrein 7 complex was detected in human plasma. Interestingly, non-inhibitory vaspinA369P was not able to improve glucose tolerance in db/db mice, clearly pointing out that this effect is critically dependent on vaspin serpin activity [14]. Moreover, in isolated murine pancreatic islets, vaspin increased insulin level after the administration of glucose but not by increasing its synthesis. Based on this finding and analysis of the crystal structure, kallikrein 7 was demonstrated as a potential target of vaspin, which prevents insulin degradation by inactivation [14]. Additionally, C-peptide levels in the supernatants and islet C-peptide content were not different between a vaspin-treated and control group, confirming that increased insulin accumulation in the medium is linked with the reduced insulin degradation [14] (Figure 3). The obtained findings clearly summarized all mechanisms of vaspin’s stimulatory effect on insulin secretion. Moreover, in vivo studies showed that vaspin treatment increased glucose tolerance and improved the insulin sensitivity in rats fed a high-fat diet, which clearly confirmed data from in vitro research [55]. In contrast to these findings on a rodent model, a study in a group of adolescents showed that, regardless of age, gender, and body mass index (BMI), lower vaspin levels were associated with better insulin sensitivity. Moreover, after oral administration of glucose, the level of plasma vaspin was significantly reduced in adolescents with insulin resistance [26]. Nevertheless, in obese children, despite the higher plasma concentration of vaspin compared to non-obese children, no significant effect of vaspin on insulin resistance was observed [72]. We suppose that the observed differences may be species related, and serum vaspin may be regulated by different factors, so the vaspin level in the serum does not fully reflect its dependence on insulin resistance. Interestingly, as noted previously, adipose tissue and liver mRNA expression are not useful predictors of vaspin serum level [29]. Hence, in target tissues, vaspin is considered an insulin-sensitizing hormone in both humans and rodents. For instance, its higher mRNA expression level was detected in the adipose tissue and skeletal muscles of obese elderly people compared to those that were lean. By activating the PI3K/AKT pathway and promoting GLUT4 translocation in obese individuals, vaspin increased the glucose uptake in skeletal muscle [60]. These results agree with other findings obtained in experiments on rats fed a high-fat diet, in which the injection of vaspin in rats’ complex led to improved insulin resistance in adipose tissue, the liver, and skeletal muscle by enhanced transduction of the IRS/PI3K/Akt/GLUT2 pathway, which simultaneously inhibited the IκBα/NFκB-signaling pathway [73]. The same findings were obtained by Shaker et al. [33]; in rats fed a high-fat diet, increased expression of *vaspin* in visceral adipose tissue was associated with increased levels of fasting serum insulin, as well as higher homeostasis model assessment of the insulin resistance index (HOMA-IR), indicating a contribution to the development of insulin resistance. Improvement in insulin sensitivity was achieved in a group of rats that additionally performed physical exercise [33]. In human vascular smooth muscle and vascular endothelial cells, through the activation of the PI3/AKT, an inhibitory effect of free fatty acid on insulin signaling was partially reversed by vaspin treatment, revealing an anti-apoptotic effect of vaspin in insulin-stimulated cells [74]. This finding comprehensively describes vaspin as a new target in diabetes treatment.

## 7. Vaspin Expression and Action in the Thyroid Gland

The relationship between thyroid gland function, insulin resistance, and obesity are of clinical interest; thus, adipokines, as a linker between them, are intensively studied. There is still little research on the relationship between the thyroid gland and expression of vaspin. In the case of patients with overt hypothyroidism, subclinical hypothyroidism, and normal thyroid function, there were no differences in vaspin levels among the groups; additionally, there was no correlation between vaspin and thyroid-stimulating hormone (TSH), T3, or T4 levels [75], but the limitation of the study was the small sample size. Moreover, some research showed that in patients with hypothyroidism, the level of vaspin in serum was significantly increased, whereas in patients with hyperthyroidism, it was significant decreased compared with the control group [76]. The observed differences between studies may be dependent on the patients’ origins. It is a well-known fact that gene polymorphism is related to population origin and may modulate protein properties. Interestingly, weight loss induced by gastric bypass surgery [77] decreased levels of vaspin and TSH in serum, but there is no conclusion in regard to whether vaspin causes a decrease in TSH levels because of weight loss or whether thyroid function influences serum vaspin levels. Future study is needed to discover vaspin expression and function in the thyroid.

## 8. Vaspin Expression and Action in the Ovaries

Vaspin and GRP78 mRNA and protein expression has been described in porcine ovary components, including ovarian follicles, oocytes, and CL. Immunolocalization was shown in granulosa and theca cells [28], and strong immunostaining was found in large and small luteal cell cytoplasm [58] as well as in cumulus cells and oocytes [78]. Moreover, the gene and protein content of vaspin depend on the oestrus cycle phase and fat level; in ovarian follicles, a higher vaspin level was detected in fat Meishan pigs compared to lean Large White pigs [28]. Conversely, in CL, vaspin and GRP78 mRNA and protein change during the luteal phase, increasing from the early luteal phase to the late luteal phase [58]. Cyclic changes in vaspin level suggested regulation by steroid hormones or gonadotrophins. Differences in vaspin and GRP78 expression were also observed in oocytes, where protein expression of both ligand and receptor increased after in vitro maturation, indicating vaspin’s influence on this process [78]. Additionally, the latest findings indicate that vaspin and GRP78 are also expressed on mRNA and protein level in tumor ovarian cell line KGN, as well as human granulosa cells; immunostaining was detected at all stages of ovarian follicular maturation [79]. Vaspin levels in the ovaries depend on local regulator factors. The ovarian follicle was stimulated by FSH, luteinizing hormone (LH), P4, E2, testosterone (T), insulin, and insulin-like growth factor type 1 (IGF1), which participates in follicle growth and development [28]. CL was negatively regulated by LH, P4, and prostaglandins (PG) PGE2 and PGF2α [58], which regulate CL formation, maintaining function and regression [8] (Figure 2). Differences in P4 and LH action in both cell types may be linked to studied structures. Depending on the luteal cycle phase and regulated by essential ovarian physiology factors, vaspin expression indicates the important role of vaspin in ovarian function control.

As an active endocrine organ, ovarian follicles synthesize steroid hormones from cholesterol due to the participation of many enzymes that control and maintain female sexual development and behavior. Data showed that vaspin in ovarian follicles stimulated basal P4 and E2 synthesis, steroidogenic acute regulatory protein (STAR), cytochrome P450 family 11 subfamily A member 1 (CYP11A1), hydroxy-delta-5-steroid dehydrogenase (HSD3B), cytochrome P450 family 17 subfamily A member 1 (CYP17A1), and cytochrome P450 family 19 subfamily A member 1 (CYP19A1) mRNA and protein expression, and decreased IGF1, FSH, and LH induced steroidogenesis by activation of the GRP78 receptor and protein kinase A (PKA) [80]. Similar to the KGN cell line and human granulosa, vaspin increased P4 and E2 secretion in basal conditions, and additionally stimulated FSH- and IGF1-induced steroid secretion by the GRP78 receptor [79]. Differences were observed between vaspin and IGF1 or FSH interactions in human and pig ovarian follicle cells, indicating that they are probably species dependent or dependent on the culture model used. In human studies, a monoculture of granulosa cells was used, whereas in the porcine model, a coculture of both granulosa and theca cells that build ovarian follicles was used, in which cooperation is needed for efficient steroidogenesis. Moreover, a stimulatory effect was observed on STAR and CYP11A1 protein expression, with no effect on HSD3B in human granulosa cells [79]. Similarly, in CL, vaspin stimulated basal P4, E2 secretion, and STAR, CYP11A1, HSD3B, and CYP19A1 proteins by GRP78 and PKA [58]. Furthermore, CL formation and regression depend on the interaction between luteotropic and luteolytic factors; PGE2 increased P4 synthesis and regulated CL formation, whereas PGF2α decreased P4 synthesis and led to CL luteolysis [8]. Vaspin upregulated the CL ratio of luteotropic PGE2 to luteolytic PGF2α and the ratio of its receptor PTGER1 to PTGFR [58]. Additionally, angiogenesis is another important process for CL function maintenance. The development of capillaries from pre-existing blood vessels is essential for the formation, function, and establishment of CL as an endocrine gland by supplying cholesterol for P4 production and regulation by vascular endothelial growth factor (VEGFA), fibroblast growth factor 2 (FGF2), and angiopoietin 1 (ANGPT1) [81]. Inappropriate vascularization leads to luteal phase deficiency and consequently miscarriages. The stimulatory effect of vaspin on VEGFA, FGF2, and ANGPT1 secretion and mRNA expression, as well as VEGFA receptor protein expression, was previously demonstrated [81]. As a molecular mechanism of vaspin regulatory action, prostaglandin synthesis and angiogenesis were shown to activate GRP78 and MAP3/1. Additionally, vaspin was reported to be a pro-survival factor for granulosa and luteal cells by increasing the ratio of B-cell lymphoma 2/ BCL2-associated X (BCL2/BAX), proliferating cell nuclear antigen (PCNA), cyclin D, and A protein and mRNA expression, and decreasing caspases 3, 8, and 9 mRNA, protein expression, and activity [59,81]. Endocrine function maintenance and regression and ovarian follicle growth and their ovulation are necessary processes for CL formation. In the KGN cell line and human granulosa cells, vaspin stimulated proliferation [79]. Finally, vaspin affected oocyte maturation, which is a critical component of embryo production in mammals and is characterized by periods of meiotic arrest and resumption; the balance between factors promoting or inhibiting oocyte maturation is necessary for subsequent fertilization and embryo development [82]. Vaspin increased porcine oocyte maturation, which was confirmed by increased cumulus-oocyte complex (COCs) P4 production via the activation of MAP3/1 and PRKAA1 pathways [78] (Figure 3).

Taken together, all these data indicate a positive effect of vaspin on ovarian functions. This adipokine may be used for oestrus cycle synchronization, to prevent luteal phase deficiency, as well as to improve in vitro fertilization protocol in particular, in order to increase the production of farm animals.

## 9. Vaspin Expression and Action in the Placenta

Vaspin expression was detected in another reproductive component, the placenta. Huo et al. [83] found the vaspin gene and protein in the human terminal placenta, whereas Caminos et al. [27] described vaspin in human and rat placentas. Furthermore, they observed species-specific immunostaining differences. Rat terminal placenta demonstrated vaspin in the trophoblast of fetal villi, the labyrinth. In humans, vaspin was localized in cytotrophoblasts and syncytiotrophoblasts in first-trimester placentas, but only in syncytiotrophoblasts in third-trimester placentas. Additionally, in humans, higher vaspin expression was observed at the end of pregnancy, suggesting a role in delivery. Food restrictions were described as a factor stimulating vaspin mRNA and protein, which suggests that these adipokines may be involved in the metabolic functions of placenta regulation [27] (Figure 2). Furthermore, as mentioned by Trojnar et al. [84], elevated vaspin levels were observed in the blood of mothers with excessive gestational weight gain, indicating that vaspin can be a new marker of lipid metabolism in pregnancy.

## 10. Vaspin Expression and Action in the Testes

Limited data indicate the role of vaspin in the testes. As shown by Brzoskwinia et al. [85], vaspin signals were localized to Leydig cells, whereas GRP78 was present in both testicular compartments in rats [85]. As the authors mentioned, a wide allocation of vaspin receptors within the seminiferous epithelium suggests its possible role in the regulation of Sertoli and germ-cell functioning. Moreover, the androgen receptor inhibitor flutamide decreased vaspin and GRP78 mRNA and protein expression in the testes [85], linking this adipokine with steroidogenesis (Figure 2). Notably, serum vaspin levels were negatively correlated with T and LH, as well as with semen quality parameters [86], indicating the inhibitory effect of vaspin on endocrine testes function. There are no data about vaspin function in the testes or its connection with testis pathologies; nevertheless, the expression of vaspin in the testes and its correlation with hormones indicate that vaspin regulates testis function, similar to other adipokines. For example, chemerin suppresses T production and HSD3B mRNA and protein level in rat Leydig cells via the MAP3/1 pathway [87]. Thus, it appears very important to investigate vaspin in term of blood and seminal plasma but also in the testis tissue in some testis pathologies.

## 11. Vaspin Connection with Endocrine Pathology

The expression of vaspin and the complex role of vaspin in endocrine tissues indicate that this adipokine may be associated with various pathologies of the endocrine system. Changes in its plasma or tissue levels can play a compensatory role in various diseases, and with other metabolic parameters it could be used as a diagnostic marker of various pathologies (Table 1). Briefly, prolactinoma, a pituitary cell cancer connected with insulin resistance caused by downregulating insulin receptors, is linked with decreased vaspin levels, which clearly links vaspin with insulin-level regulation in the body [88].

Next, MetS is a group of interconnected elements characterized by hypertension, abdominal obesity, dyslipidemia, glucose intolerance, and insulin resistance. Multiple authors indicated vaspin as a predictor of MetS; a high serum vaspin concentration could be used as a diagnostic marker of MetS in adults [89] and children [90]. Other authors indicated a correlation between vaspin and some components of MetS, such as insulin resistance and chronic inflammation [91]. Some inconsistency was noted in vaspin levels and MetS in men’s serum. Higher levels were detected by Choi et al. [92], whereas Kim et al. [93] described downregulation in vaspin serum level [93]. Additionally, vaspin was negatively correlated with waist circumference, systolic and diastolic blood pressure, serum triglyceride level, and abdominal VAT [93], and we suppose the observed differences were dependent on vaspin correlations with these parameters. Nevertheless, vaspin may be an important predictor of MetS in humans, facilitating a quick diagnosis, especially taking into account the correlation of its plasma level with other parameters characterizing MetS. Additionally, some findings indicate that fenofibrate, a clinical treatment of dyslipidemia and insulin resistance, increases vaspin mRNA and protein expression in retroperitoneal adipose tissue (RET), mesenteric adipose tissue (MET), and epididymal adipose tissue (EAT) without the influence of *vaspin* mRNA in subcutaneous adipose tissue (SAT), which works as a compensatory mechanism in MetS [94]. Moreover, the latest findings indicated that vaspin gene polymorphism may be associated with MetS; the TA genotype of vaspin rs2236242 was related to a greater risk of MetS and its components, which was reversible after 60 min of walking [95].

Vaspin is also involved in pathophysiology associated with impaired insulin signaling, as well as insulin sensitivity. In patients with abdominal obesity after acute pancreatitis, a positive correlation was demonstrated between the levels of vaspin and insulin resistance [96]. Vaspin has been described in T2DM. For instance, Wistar rats with diabetes induced via a high-fat diet had lower levels of vaspin than the non-diabetic group [97]. In contrast, in people with T2DM, a higher concentration of vaspin was observed compared to the control group [98,99,100]. In this group of patients, the level of vaspin significantly correlated with BMI, waist-to-rip ratio (WHR), and the level of triglycerides and insulin in the fasting plasma [98]. Studies among a Chinese population showed that patients with T2DM had a higher incidence of the homozygous mutant *vaspin* genotype. This marked polymorphism of the vaspin rs2236242 gene may correlate with the development of T2DM. Interestingly, no relationship was found with the development of obesity [101]. The increasing concentration of vaspin in people with TDM may be a risk factor for the development of coronary artery disease [99]. Nevertheless, the opposite results were obtained by Jian et al. [102] in their two-year cohort study examining the progression of T2DM in patients. They observed that the concentration of plasma vaspin was lower in the diabetic group compared to the control. Additionally, the authors associated the lower plasma concentration of vaspin with increasing disease progression [102]. This result may depend on long diabetes duration. Long insulin treatment may affect the plasma vaspin levels and thus induce the decrease in vaspin. Additionally, the control subjects were patients with hypertension, so plasma vaspin level may be modulated with different drug treatments. Youn et al. [103] did not find a correlation between circulating vaspin and BMI in patients with T2DM. However, in the normal glucose-tolerant group, circulating vaspin significantly correlated with BMI and insulin sensitivity [103]. Notably, insulin resistance and diabetes are often associated with both local and systemic inflammation. Therefore, the ability of vaspin to suppress inflammatory responses is also considered here as beneficial because it indirectly prevents the development of such disorders. In several studies on potential drugs improving insulin sensitivity in T2DM, vaspin, like other adipokines such as omentin, visfatin, or adiponectin, is considered a biomarker of diabetes and a potential indicator of drug efficiency [104,105].

Vaspin has also been linked to medullary thyroid carcinoma (MTC), which is a type of thyroid cancer [106]. Patients with MTC had significantly higher levels of vaspin in serum compared to the control group, and elevated vaspin levels are associated with an increased risk of MTC [106]. Based on recent studies, vaspin has anti-apoptotic effects that could indicate a relationship between cancer development and vaspin expression, and future studies are needed to understand the mechanism of vaspin action in MTC.

Literature data also indicate a connection between vaspin and PCOS, the most common ovarian disorder. This syndrome was characterized by Stein and Leventhal in 1935. They described women with hirsutism, obesity, and ovaries covered with numerous cysts [107]. The first correlation of vaspin with PCOS was mentioned by Tan et al. [13] in 2008, who showed elevated vaspin plasma levels in PCOS patients. A similar correlation was observed by Cekmez et al. in 2011 [108]. Moreover, higher vaspin concentrations were independently correlated with BMI and a homeostasis model assessment of insulin resistance [66]. Likewise, the results observed by Cakal et al. [109] indicate that serum vaspin could differentiate between women with and without increased diabetogenic risk [109]. Interestingly, significantly upregulated vaspin transcripts and protein were found in the omental adipose tissue of women with PCOS, and the stimulatory effect of glucose on vaspin expression was demonstrated by Tan et al. [13]. Furthermore, six-month metformin treatment significantly decreased serum vaspin levels in women with PCOS [13]. Additionally, Bongrani et al. [79] showed that vaspin levels in both follicular fluid and granulosa cells increased in obese women and were positively correlated with BMI, whereas the GRP78 level was constant [79]. Furthermore, weight-dependent vaspin concentration was studied; higher concentrations were observed in normal-weight patients with PCOS than normal-weight controls, but plasma vaspin levels were not significantly increased in overweight/obese patients with PCOS compared to overweight/obese controls [66]. Increased plasma vaspin levels might represent a compensatory mechanism to preserve insulin sensitivity and glucose tolerance, which are impaired in obesity. Conversely, no difference in vaspin plasma levels was observed by Akbarzadeh et al. [110] and Guvenc et al. [111]. Interestingly, in Guvenc et al.’s study, the patients in the PCOS group did not have insulin resistance, which may explain the lack of effect on vaspin levels. Notably, Dogan et al. [112] compared serum vaspin in women with PCOS in groups with failed ovulation induction and successful ovulation induction and measured significantly lower vaspin levels in responders achieving ovulation, indicating this adipokine as a predictor of ovulation in PCOS patients [112]. Furthermore, the effect of physical activity on PCOS patient conditions was intensively studied, but, as described, interval training did not change vaspin levels in PCOS subjects [113]. The presented data show that due to the close link between vaspin and PCOS, this adipokine can be a clinical marker in the diagnosis of present abnormalities.

Vaspin influences placenta/pregnancy pathology, as previously described. Gestational diabetes mellitus (GDM) occurs in 1–14% of pregnant women and is described as glucose intolerance and chronic insulin resistance discovered with the onset or first recognition of pregnancy. Significantly lower vaspin serum concentrations were observed in the GDM group compared to the control, but no differences in placenta mRNA or protein expression were detected. Conversely, in GDM, placenta vaspin content negatively correlated with neonatal birth weight [83]. Similar data were obtained by Mierzynski et al. [114]. In contrast, Mm et al. [115] and Tang et al. [116] observed significantly higher vaspin concentrations in GDM than in the control group, in addition to upregulated vaspin gene and protein expression in SAT and VAT [116]. Conversely, Stepan et al. [117] and Gkiomisi et al. [118] claimed that serum vaspin cannot independently predict GDM and is not affected by the degree of glucose metabolism dysregulation [118]. These data indicate the possible involvement of vaspin in GDM, but the interpretation of results is difficult due to numerous limitations, such as the diagnostic criteria for GDM, the gestational age of evaluation, and methods of analysis, which are widely different between studies [119]. Next, IUGR is characterized by fetal growth that is less than normal for the population and less than the growth potential of that infant [120]. Cord vaspin at birth was higher in small-for-gestational-age fetuses compared to the control, whereas at five days postnatal, differences in vaspin concentration were not observed [121]. As the authors suggested, adulthood diseases originated from the adaptation that the fetus makes when it is undernourished, connecting high vaspin levels with an increased risk for adult metabolic diseases. Future studies are needed to confirm these data. However, this knowledge indicates the possibility to use vaspin as a marker of reproductive pathology.

## 12. Perspectives

In the present paper, the findings indicate the clinical application of vaspin in medicine, especially in endocrinology, as well as in reproduction. Vaspin may be used to prevent obesity due to its inhibitory action on the orexigenic pathway in the brain [24]. Data indicate that vaspin may be a new drug designed to treat diabetes due to its insulin-sensitizing effect in WAT and inhibitory properties on gene expression associated with insulin resistance [11], as well as its stimulatory effect on insulin secretion [55]. The modulatory effects of vaspin described in the reproductive tract indicate that vaspin regulates proliferation and steroidogenesis of ovarian follicle cells, demonstrating its action in folliculogenesis and ovulation. Thus, vaspin could be used to improve oestrus/menstrual cycle synchronization and to enable reproductive optimization [59,80]. Additionally, it is a luteotropic factor in luteal cells, which may prevent luteolysis of CL and luteal phase deficiency, causes of infertility and miscarriages [58,81]. Vaspin also improved oocyte maturation [78], which in the future may refine in vitro fertilization protocols. This adipokine probably regulates male germ-cell steroidogenesis [85]. The data describing vaspin function in ovarian and testicular cells will be useful to regulate agricultural species, including pigs breeding. Finally, some authors indicated vaspin as a clinical predictor and novel biomarker of many pathologies, including GDM, PCOS [13], MetS [89], hypo- and hyperthyroidism [76], MTC [106], and semen quality [86].

## 13. Conclusions

In conclusion, vaspin is expressed in endocrine tissues, including the hypothalamus, pancreas, adipose tissue, ovaries, placenta, and testes. Its action in the organism relates to the regulation of insulin sensitivity, preadipocyte differentiation, steroid synthesis, and oocyte maturation, in addition to the described adipokine functions, such as regulation of proliferation and apoptosis and CL formation, angiogenesis, and regression. Finally, there was an observed connection between vaspin and different pathologies, including diabetes, MTC, MetS, PCOS, GDM, and IUGR. Knowledge about vaspin action in endocrine tissues is important to properly understand whole-body physiology and can prevent diabetes or infertility events connected with the described abnormalities. In addition, a lack of data about vaspin action in the adrenal gland, as well as limited information in testis function, leaves a wide scope for future research in this area. This may lead to a better understanding of events, such as reproduction and fertility.

## Figures and Tables

**Table 1:** Vaspin serum-level association with endocrine pathologies. ↑—increase; ↓—decrease, ns—no study.

Pathology	Species	
	Rat	Human
Prolactinoma	ns	↓
Metabolic syndrome	↑	↑; ↓
Diabetes	↓	↑; ↓
Hypothyroidism	ns	↑
Hyperthyroidism	ns	↑
Medullary thyroid carcinoma	ns	↑
Polycystic ovarian syndrome	ns	↑
Gestational diabetes mellitus	ns	↑; ↓
Intrauterine growth restriction	ns	↑

## Data Availability

Not applicable.
